# An improved SOM algorithm and its application to color feature extraction

**DOI:** 10.1007/s00521-013-1416-9

**Published:** 2013-04-27

**Authors:** Li-Ping Chen, Yi-Guang Liu, Zeng-Xi Huang, Yong-Tao Shi

**Affiliations:** 1 Lab of Vision and Image Processing, College of Computer Science, Sichuan University, Chengdu, 610065 China; 2 College of Information and Engineering, Tarim University, Alaer, 843300 China

**Keywords:** Self-organizing map, Color feature extraction, Non-principal component, Competitive mechanism

## Abstract

Reducing the redundancy of dominant color features in an image and meanwhile preserving the diversity and quality of extracted colors is of importance in many applications such as image analysis and compression. This paper presents an improved self-organization map (SOM) algorithm namely MFD-SOM and its application to color feature extraction from images. Different from the winner-take-all competitive principle held by conventional SOM algorithms, MFD-SOM prevents, to a certain degree, features of non-principal components in the training data from being weakened or lost in the learning process, which is conductive to preserving the diversity of extracted features. Besides, MFD-SOM adopts a new way to update weight vectors of neurons, which helps to reduce the redundancy in features extracted from the principal components. In addition, we apply a linear neighborhood function in the proposed algorithm aiming to improve its performance on color feature extraction. Experimental results of feature extraction on artificial datasets and benchmark image datasets demonstrate the characteristics of the MFD-SOM algorithm.

## Introduction

Despite much progress in the field of feature extraction in recent years, achieving robust and effective features remains a challenging problem. The self-organizing map (SOM) [1–3], which is known as an unsupervised learning algorithm, has been widely and successfully applied to many problem domains, such as speech recognition, image and video processing [4–9]. In those applications, characteristics of SOM algorithm, including feature extraction, vector quantization, dimension reduction, and topology preservation, play important roles. However, based on the thought that an algorithm should extract as much and as accurate description as possible to the training data, features extracted by conventional SOM algorithm are usually redundant, especially the features extracted from the principal components in the training data. Moreover, some heuristic or discriminative features could be represented coarsely, or even lost, after learning just because of their lower ratios in the training data. Therefore, it is of great interest to improve conventional SOM to extract more robust and diverse features.

To a certain degree, the quality of features extracted by the SOM algorithm depends on the neighborhood function, which determines the local distribution of weight vectors of neurons in the lattice. Generally, four types of neighbor functions are available, including “bubble”, “Gaussian”, “cut-gauss”, and “ep” (or Epanechikov function) [10]. Different from those symmetric neighborhood functions, Aoki and Aoyagi [11] proposed an asymmetric neighborhood function to accelerate the ordering process of SOM algorithm.

For the purpose of visual display, the rectangular or hexagonal lattice is frequently used in practice. Due to the well-known problem called boundary or edge effect for such irregular network topologies as rectangular or hexagonal topologies, regular hyperbolic and spherical topologies satisfying each neuron owns an equal number of neighbors in the lattice have also been studied [12–15]. Additionally, to overcome the limitations of the static network structure, dynamic and growing lattices have been applied to fields like automatic organizing of documents and knowledge discovery [16–18]. Inspired by the regular hyperbolic and spherical topologies, a simplified regular rectangular lattice is adopted in the proposed algorithm.

Early evaluations of the performance of the SOM algorithm mainly focus on comparisons with other techniques, such as principal component analysis and k-means clustering [19]. However, recently, Kohonen et al. [20] figured out that this kind of comparison was usually taken as self-evident. After careful and systematic examination, they found that results of comparisons depend most strongly on the ratio of the number of training vectors and the number of model vectors. Their conclusion reveals important factors acting on the competitive learning.

In order to improve the computational performance of the SOM algorithm, several variants, such as the conscience and the batch-update algorithms, have been proposed [1, 2, 21]. Although much progress have been made, the expensive time consumed by the SOM algorithm still hinders its application to such fields as data mining and image processing, where the training data are huge, and the improvement achieved by common methods are usually not obvious.

Self-organizing map networks have been used for image processing in some previous studies. To reduce the number of colors of an image with minimum distortion, color reduction has been studied [6, 22, 23]. Due to not providing extra protection for non-dominant colors of an image, color reduction may lead to weakening or missing some non-dominant but necessary colors in the process. Recent researches in [7, 24, 25] show the popularity of SOM in various image processing problems.

This paper focuses on improving the SOM algorithm to efficiently extract more robust color features from images. The rest of this paper is organized as follows. In Sect. 2, we introduce the conventional SOM algorithm and then explain the proposed algorithm. In Sect. 3, details of the proposed algorithm and its optimization on color feature extraction are presented. Experimental results are introduced in Sect. 4. Main conclusions and discussions are made in the last section.

## Methods

### Self-organization map

The network of conventional SOM usually consists of n × m neurons located at a two-dimensional rectangular or hexagonal grid. Each neuron *i* has a *d*-dimensional weight vector *w*_*i*_ = (*w*_*i*1_, *w*_*i*2_, …, *w*_*id*_) (*i* = 1, 2, …, *nm*). The initial values of all the weight vectors are given over the input space at random. The range of the elements of *d*-dimensional input data *x*_*j*_ = (*w*_*j*1_, *w*_*j*2_, …, *w*_*jd*_) (*j* = 1, 2, …, *N*) are assumed to be from 0 to 1.

When a training vector *x*_*j*_ is fed to a network, a winner *c*, is the neuron with the weight vector closest to the training vector *x*_*j*_, which can be denoted as

1 $$ c = \arg \mathop {\hbox{min} }\limits_{i} \left\{ {\left\| {w_{i} - x_{j} } \right\|} \right\}, $$

where ||·|| is the Euclidean distance.Then, the weight vectors of the winner and its neighbors can be updated as

2 $$ w_{i} \left( {t + 1} \right) = w_{i} \left( t \right) + h_{c,i} \left( {x_{j} - w_{i} \left( t \right)} \right), $$

where *t* is the learning step. *h*_*c*,*i*_(*t*) is called the neighborhood function and is described as

3 $$ h_{c,i} = \alpha \left( t \right)\exp \left( { - \frac{{\left\| {r_{i} - r_{c} } \right\|^{2} }}{{2\sigma^{2} \left( t \right)}}} \right), $$

where ||*r*_*i*_ − *r*_*c*_|| is the distance between the map nodes *c* and *i* on the map grid, *α*(*t*) is the learning rate, and *σ*(*t*) corresponds to the width of the neighborhood function. Both *α*(*t*) and *σ*(*t*) decrease with time. In this study, we use the following equations, respectively

4 $$ \alpha \left( t \right) = \alpha_{0} \left( t \right)\exp \left( { - t/T_{\hbox{max} } } \right) $$

and

5 $$ \sigma \left( t \right) = \sigma_{0} \left( t \right)\left( {1 - t/T_{\hbox{max} } } \right). $$

From Eqs. (3)–(5), it can be seen that the *h*_*c*,*i*_ is a decreasing function, which is mainly dependent on the iteration time *t* but not the training error ‖*W*_*i*_ − *x*_*j*_‖. That fact decides that same learning rate and same neighborhood radius in each training epoch are indiscriminately used to achieve features of different components in the training data, which is not conductive to obtain better quality of features of non-principal components due to their competitive disadvantages.

Additionally, competition or learning directed by the conventional SOM, which uses the winner-take-all competitive principle, is of benefit to principal components due to their greater ratios in the training data. Thus, more neurons will learn features of the principal components, and consequently, those features are commonly redundant, which could deteriorate the performance of the algorithm and result in the over-fitting problem. Sensitivity tests of tunable SOM parameters including map size (number of neurons) can be seen in Liu et al. [19].

It is believed that by adding more neurons to the network, the quality of features extracted from non-principal components in training data will be improved significantly. However, such approach not only reduces the effectiveness of features due to achieving many more redundant features, but also further deteriorates the performance of conventional SOM algorithm because more iterations are required.

### Dynamic neighborhood radius and learning rate

Within the *t*-th training epoch in the conventional SOM, both values of *α*(*t*) and *σ*(*t*) are fixed for different winners in competition, namely all winners are treated equally within a training epoch. While in our algorithm, the learning rate and neighborhood size for a winner of competition are dynamic, which can be calculated by

6 $$ \alpha_{c} \left( t \right) = \alpha \left( t \right)\left( {1 - \varepsilon \exp \left( { - K_{c} \delta_{c,j} } \right)} \right) $$

and

7 $$ \sigma_{c} \left( t \right) = \sigma \left( t \right)\left( {1 - \varepsilon \exp \left( { - K_{c} \delta_{c,j} } \right)} \right), $$

where *α*(*t*) and *σ*(*t*) can be calculated by Eqs. (4) and (5), respectively, *ɛ* $(\varepsilon \in [0, 1])$ and *K*_*c*_ are user-defined constant values used to tune the learning rate and neighborhood radius for a winner. *δ*_*c*,*j*_ is the distance between *x*_*j*_ and *w*_*c*_, which can be represented as

8 $$ \delta_{c,j} = \frac{{\left\| {w_{i} - x_{j} } \right\|^{2} }}{d}. $$

It can be seen from Eqs. (6) and (7) that the learning rates [*α*_*c*_(*t*)] and neighborhood radiuses [*σ*_*c*_(*t*)] for winners of competition are dynamic within each training epoch, which partly depends on the value of *δ*_*c*,*j*_. If *δ*_*c*,*j*_ → 0, then *σ*_*c*_(*t*) ≈ *σ*(*t*)(1 − *ɛ*) and *α*_*c*_(*t*) ≈ *α*(*t*)(1 − *ɛ*), a weakened learning for the input *x*_*j*_ occurs. On the contrary, if *δ*_*c*_ → 1 and *K*_*c*_ is a big constant, then *σ*_*c*_(*t*) ≈ *σ*(*t*) and *α*_*c*_(*t*) ≈ *α*(*t*), a learning for *x*_*j*_ will be normal. Compared with the weakened learning, within a training epoch, if a normal learning occurs, the winner of competition can be given a larger neighborhood radius and a greater learning rate.

Being of greater ratios in the training data, features of the principal components in the training data will be ahead of non-principal components to be trained well. Thus, the normal learning is mainly triggered by non-principal components in the training data. Compared with the conventional SOM, features of non-principal components are given more chance to compete, and thereby better representations of their features can be achieved by the proposed algorithm. What is more, with the decrease of training error, the number of neurons needed to update will also decrease, which is benefit to improve the performance of the proposed algorithm.

**Fig. 1:**
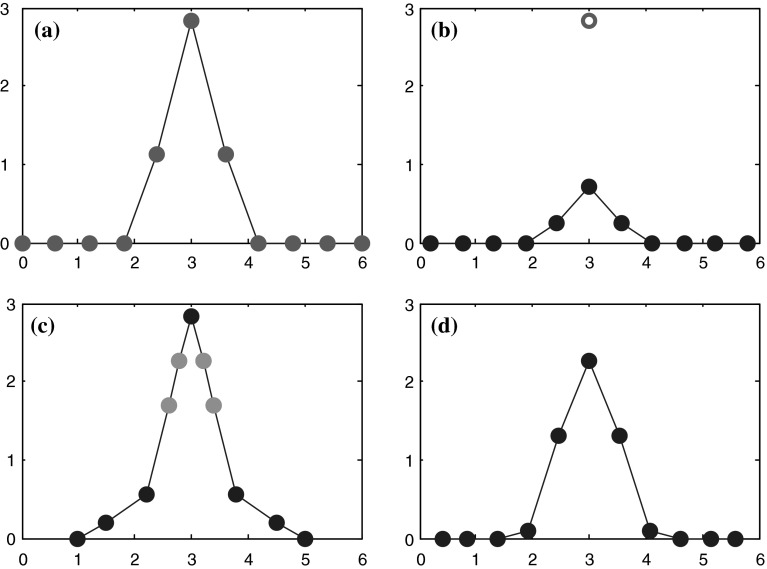
Demonstrations of two kinds of competitions. **a** Virtual training samples. **b** A possible result trained by the conventional SOM. **c** A possible intermediate result trained by MFD-SOM. **d** A possible final result trained by MFD-SOM (color figure online)

Figure 1 demonstrates the different competitive behaviors of both algorithms. In Fig. 1a, coordinates (x_i_, y_i_) of the eleven red points are used to train both networks. Figure 1b presents a possible distribution of points (x_i_, y_i_) trained by the conventional SOM. It can be seen that there exists a large distance between the sixth sample point (marked with a red circle in Fig. 1b) and the closest trained point (the sixth blue point in Fig. 1b). As to our algorithm, when it learns the position vector of the sixth sample point (red circle in Fig. 1b), due to the large training error, the learning will be normal. Thus, a possible intermediate result trained by our algorithm is demonstrated in Fig. 1c. When the training finishes, a possible distribution of points can be demonstrated by Fig. 1d. From Fig. 1d, it can be seen that the maximum distance or training error is greatly less than that achieved by the conventional SOM.

### New way to update weight vectors of neurons

To reduce redundancy of features extracted from the principal components and to avoid the problem of over-fitting, we present a new way to update weight vectors of neurons.

**Fig. 2:**
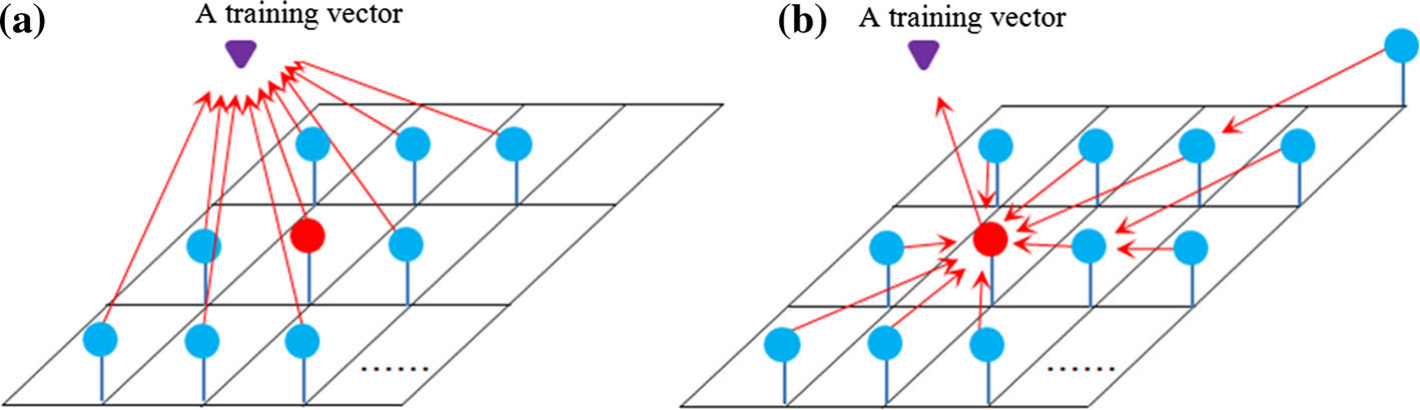
Two ways of updating weight vectors of neurons. **a** Traditional way. **b** Proposed way (color figure online)

As shown in Fig. 2a, in the traditional way, not only the winner (marked with red ball) but also its neighbors (marked with blue balls) will be directly affected by the training vector (marked with purple triangle). As to the new way, as illustrated in Fig. 2b, each neighbor of a winner will only be directly affected by the neuron closest to both the winner and itself (not including itself) in the lattice.

The weight vector (*w*_*c*_) of the current winner *c* can be directly updated by

9 $$ w_{c} \left( {t + 1} \right) = w_{c} \left( t \right) + \alpha_{c} \left( t \right)\left( {x_{j} - w_{c} \left( t \right)} \right). $$

For each neighbor of the winner *c*, its learning rate [*α*_*i*_(*t*)] is determined by

10 $$ \alpha_{i} \left( t \right) = \alpha \left( t \right)\left( {1 - \varepsilon \exp \left( { - K_{c} \delta_{i,t} } \right)} \right), $$

where *δ*_*i*,*t*_ is the Euclidean distance between both weight vectors of the neighbor and the training data.

Then, new weight vector for each neighbor of the winner can be represented as

11 $$ w_{i} \left( {t + 1} \right) = w_{i} \left( t \right) + \alpha_{i} \left( t \right)\left( {w_{t} \left( t \right) - w_{c} \left( t \right)} \right), $$

where *w*_*t*_(*t*) is the weight vector of the training data, and it should be updated before its output. By tuning the learning rate *α*_*i*_(*t*), the distance between weight vectors of the training data and its output will be neither too close nor too far. Thus, features extracted from the principal components in the training data by our algorithm will be of lower redundancy.

## Algorithm and optimization for color feature extraction

### Algorithm

**Fig. 3:**
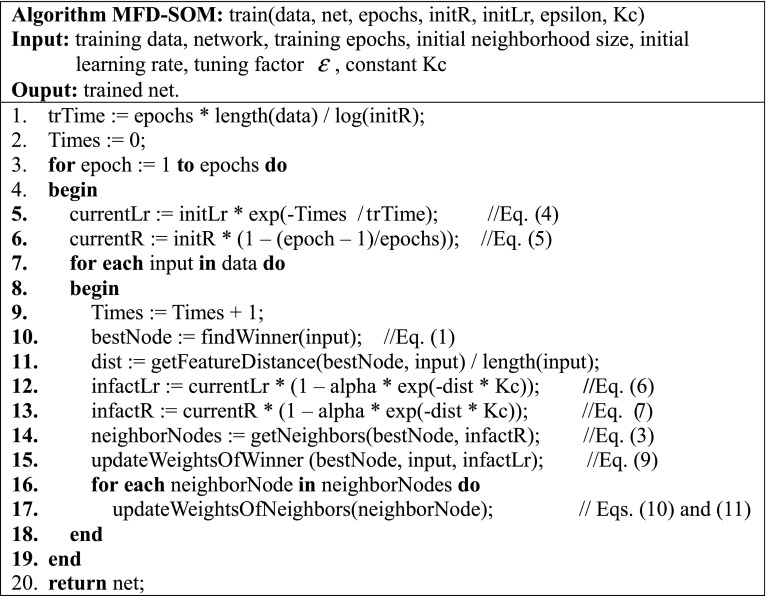
Framework of the MFD-SOM algorithm (color figure online)

The implementation of the proposed algorithm is outlined in Fig. 3, where seven input parameters are required and the output is the trained net.

### Optimization on color feature extraction

Although the SOM algorithm has achieved many successful stories [19], its application may still become infeasible when computation time is taken into account, especially when dealing with large volumes of data. The same occurs when we applied the algorithm to extract color features from images.

The performance of the SOM can be improved significantly by applying such measures, including fewer training epochs and samples. However, these measures are likely to degrade the quality of extracted features. In fact, both the performance of the algorithm and the quality of features depend on the competitive or selective mechanism of the algorithm, which decides the efficiency and effectiveness of learning and decides the preference for what kinds of features.

Due to the new competitive mechanism of the proposed algorithm, even using the linear neighborhood function (see Eq. 5) and given few training epochs, the quality of color features extracted by our algorithm is comparable or much better than those achieved by the conventional SOM (see Tables 1, 2).

**Table 1:** PSNR data of images reconstructed by the conventional SOM

Map size	PSNR (dB)					
	River	Flower	Bird	Cloth	Girl	Penguin
11 × 11	32.2447	37.2195	44.2525	48.0592	44.0169	34.8381
12 × 12	31.9429	37.7923	44.3713	48.6486	44.6506	34.9522
13 × 13	32.4071	38.4297	44.7146	49.5391	45.3980	34.9712
14 × 14	32.4495	38.9489	45.1581	50.1424	45.4908	34.9976
15 × 15	32.4819	39.8768	45.3802	50.1898	46.6564	35.0142
16 × 16	32.5203	40.2076	45.7293	50.8497	47.1776	35.0424
17 × 17	32.5526	40.8931	45.7260	51.2752	47.7313	35.0670

**Table 2:** PSNR data of images reconstructed by the MFD-SOM

Map size	PSNR (dB)					
	River	Flower	Bird	Cloth	Girl	Penguin
11 × 11	39.6985	38.0360	44.1833	46.6237	41.4816	44.0358
12 × 12	40.2204	38.6639	44.7157	47.0018	42.0940	44.5453
13 × 13	40.7778	39.0684	44.8387	47.5230	42.5389	44.9878
14 × 14	41.2790	39.5883	45.1811	47.2669	43.0675	45.6241
15 × 15	41.7602	40.0835	45.4762	47.7132	43.4795	45.9781
16 × 16	42.1252	40.4612	45.4444	47.7711	43.9299	46.4039
17 × 17	42.5293	40.7527	45.2668	48.3080	44.3671	46.5178

## Experiments and applications

In this section, we conduct experiments to demonstrate the efficiency and effectiveness of our algorithm, which has been implemented in C and Matlab languages. For doing comparison, as a reference implementation of the conventional SOM algorithm, the SOM toolbox integrated into the Matlab product (Version 2011A) is selected as a rival to our algorithm.

### Feature extraction from two artificial datasets

**Fig. 4:**
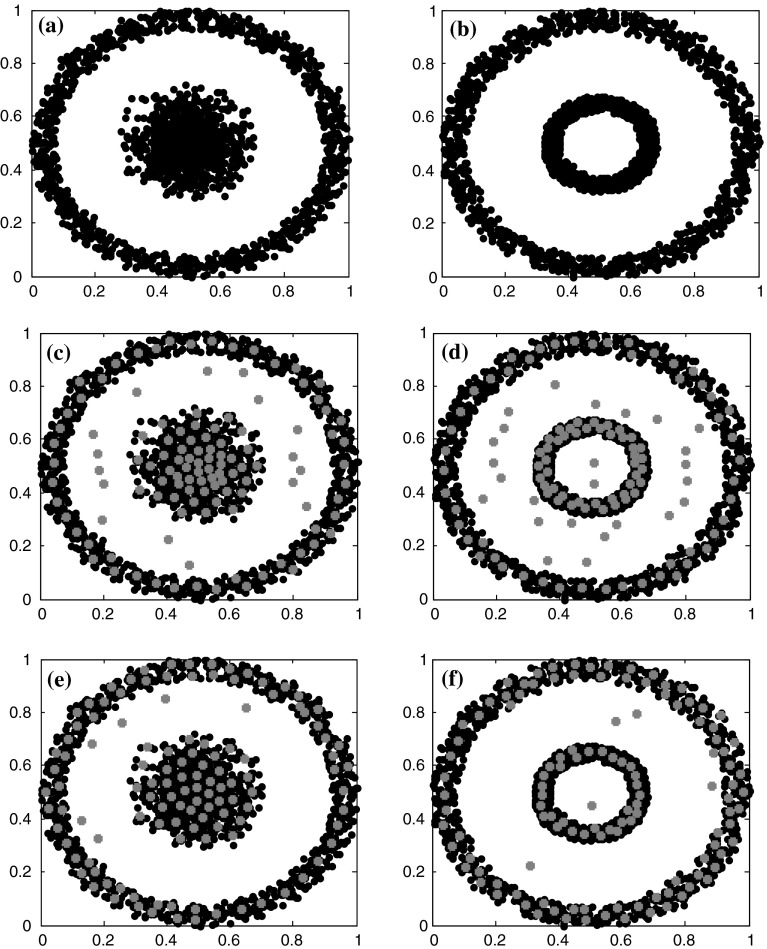
Two artificial datasets and features extracted from them by both algorithms. **a** Two artificial datasets. **b** Features extracted by the conventional SOM. **c** Features extracted by our algorithm

Two artificial datasets were generated, and each dataset has 3,000 sample points. The distributions of those points are presented in Fig. 4a, b. The Gaussian neighborhood function (see Eq. 3) was used in this experiment for our algorithm. The map sizes are 11 × 11 neurons for both algorithms. Parameters used in our algorithm, including maximum training epochs, initial radius, initial learning rate, epsilon and deltaK, are set to 300, 6, 0.3, 0.5 and 5, respectively. For the conventional SOM, the maximum training epochs, topology function, distance function, initial neighborhood size are set to 300, “hextop”, “linkdist” and 3, respectively. Figure 3c–f present features (marked with red points) extracted by both algorithms.

Comparison of distributions of red points shown in Fig. 4c–f, we can see that the conventional SOM extracted more features from those components with higher densities in training data, while our algorithm yielded more evenly distributed patterns. It can be said that our algorithm provides non-principal components in training data with protections to a certain degree. Besides, from Fig. 4c, we can see that distances between adjacent features are almost equal, and no adjacent features are too close, which demonstrate the effect of the new way to update weights of the MFD-SOM.

The scattered points between the inner and outer circles (Fig. 4b, c) are artifacts of the SOM algorithms, because they are not seen in the input data (Fig. 4a) at all. These artifacts are usually transitional features between two distinct extremes [19]. Their frequencies of occurrence are zeros, according to an experiment by Liu et al. [19] (See their Fig. 11). It is interesting that the number of the artifacts is largely reduced in the MFD-SOM results (Fig. 4c) than in the conventional SOM results (Fig. 4b). This demonstrates the improvement of the MFD-SOM over the conventional SOM in feature extraction.

### Color feature extraction from images

**Fig. 5:**
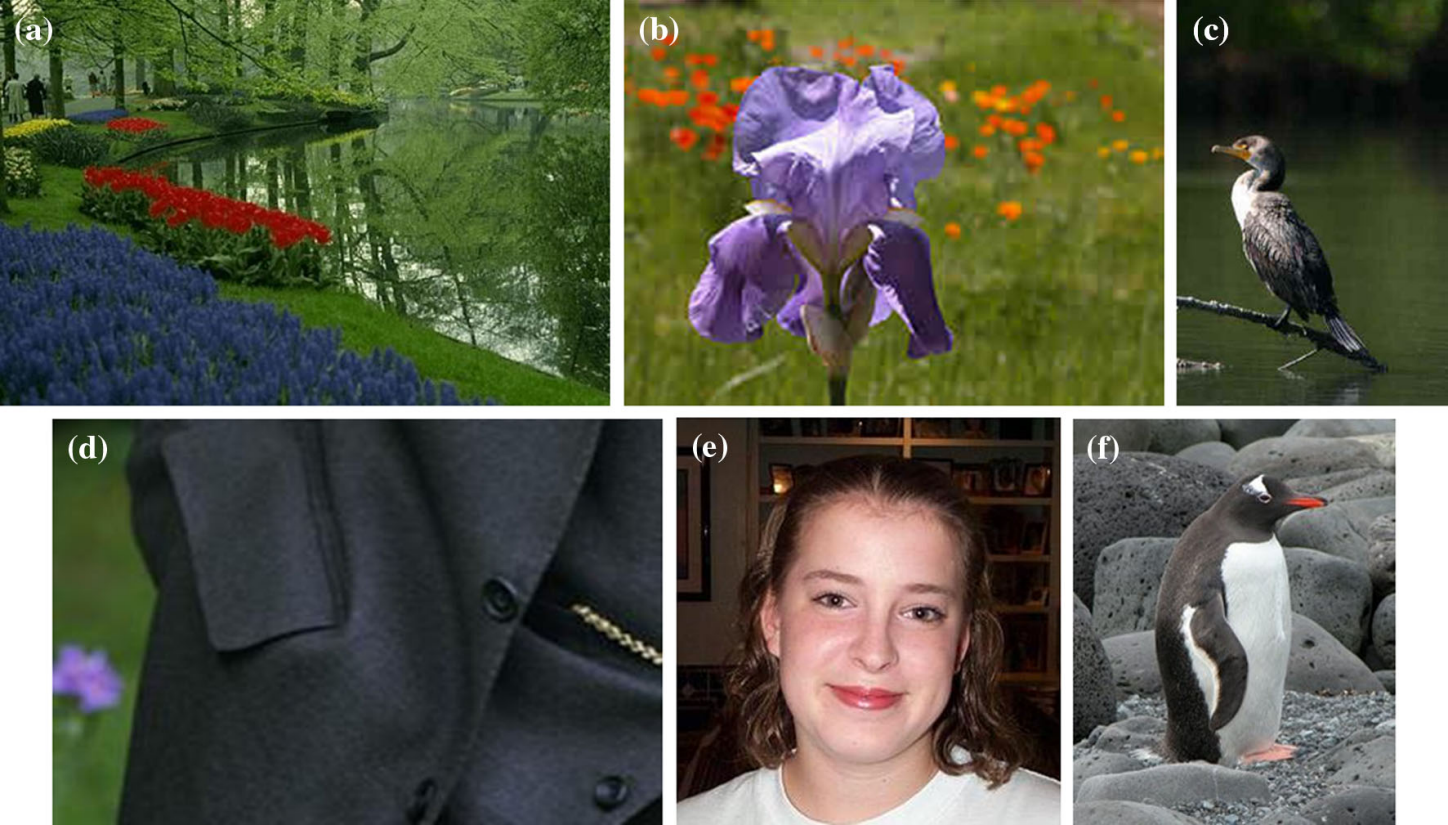
Training images. **a** River (140055.jpg). **b** Flower (118_0081.jpg). **c** Bird (049_0097.jpg). **d** Cloth (257_0178.jpg). **e** Girl (253_0354.jpg). **f** Penguin (158_0135.jpg)

**Fig. 6:**
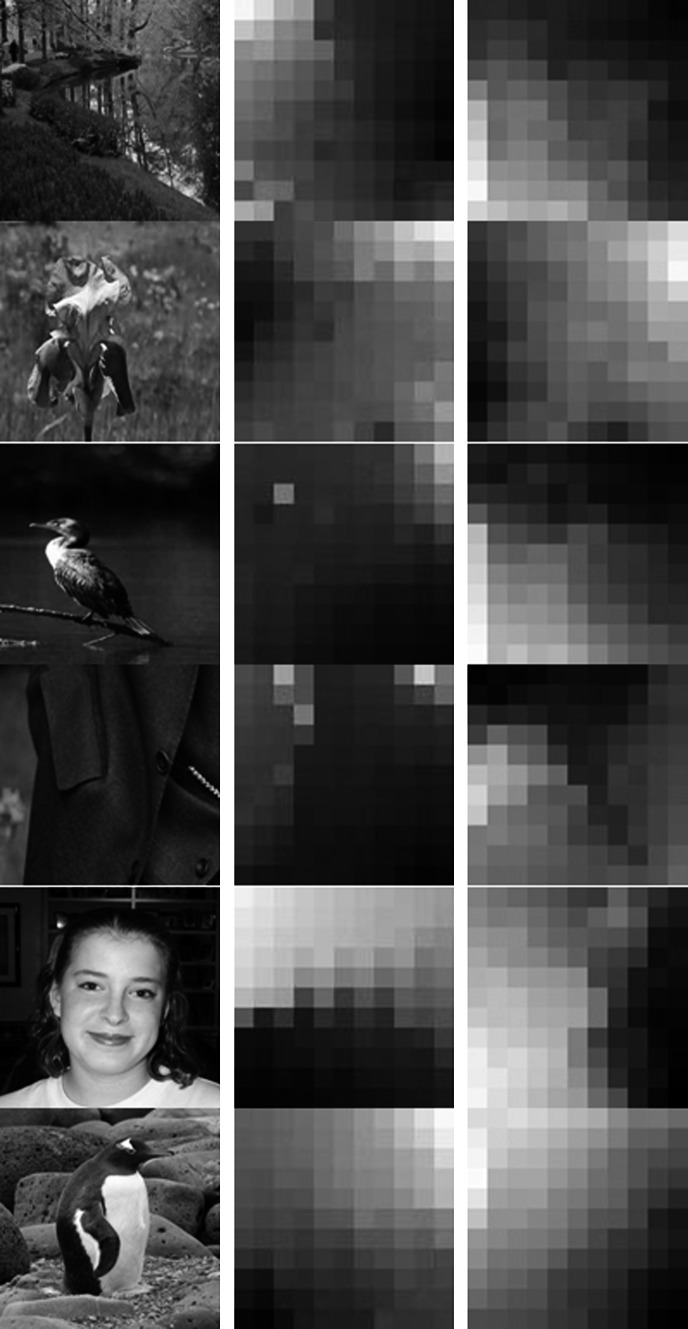
Demonstrations of extracted color features. Thumbnails of training images (*in the left column*), color features maps achieved by the conventional SOM algorithm (*in the middle column*) and achieved by our algorithm (*in the right column*)

Experiments on color feature extraction from six images from [26, 27], as shown in Fig. 5, were carried out. The map sizes are 11 × 11 neurons for both algorithms. For the conventional SOM, the maximum training epochs, topology function, distance function, initial neighborhood size are set to 200, “hextop”, “linkdist” and 3, respectively. The linear neighborhood function (see Eq. 5) is used in our algorithm to shorten the learning time for color feature extraction. Parameters used in our algorithm, including maximum training epochs, initial radius, initial learning rate, epsilon and deltaK, are set to 20, 6, 0.3, 0.5, and 100, respectively. The self-organization maps of color features, which are achieved by both algorithms and magnified by 10 times, are shown in Fig. 6.

**Fig. 7:**
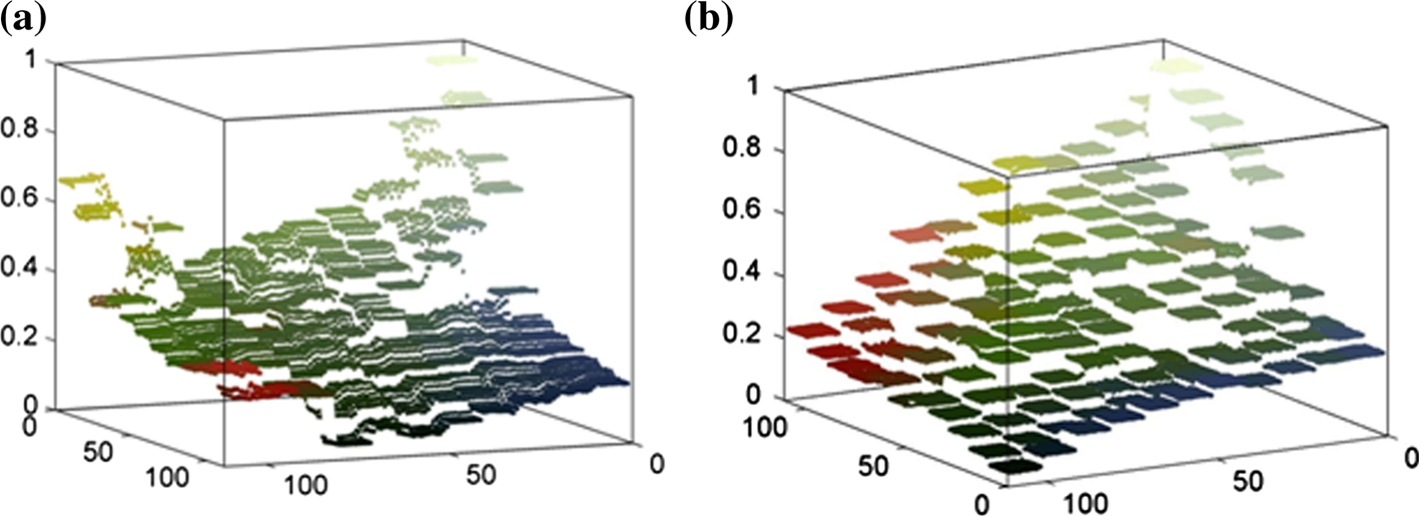
3D visualizations of colors achieved by both algorithms from the “river” image. **a** Colors extracted by the conventional SOM. **b** Colors achieved by the proposed SOM (color figure online)

Compared with the conventional SOM, it is apparent that our algorithm can better represent non-principal colors and reduce the redundancy of principal colors in training images. To demonstrate the difference between colors extracted by both algorithms, 3D visualizations of the two color maps of the “river” image are presented in Fig. 7.

From Fig. 7a, it can be seen that green color is of greater ratio in extracted colors, and the distances between them are very close. While Fig. 7b shows that the distances between same kinds of colors are mean or approximately equal. Thus, the goal of reducing the redundancy of features of the principal components in the training data is reached by our algorithm.

**Fig. 8:**
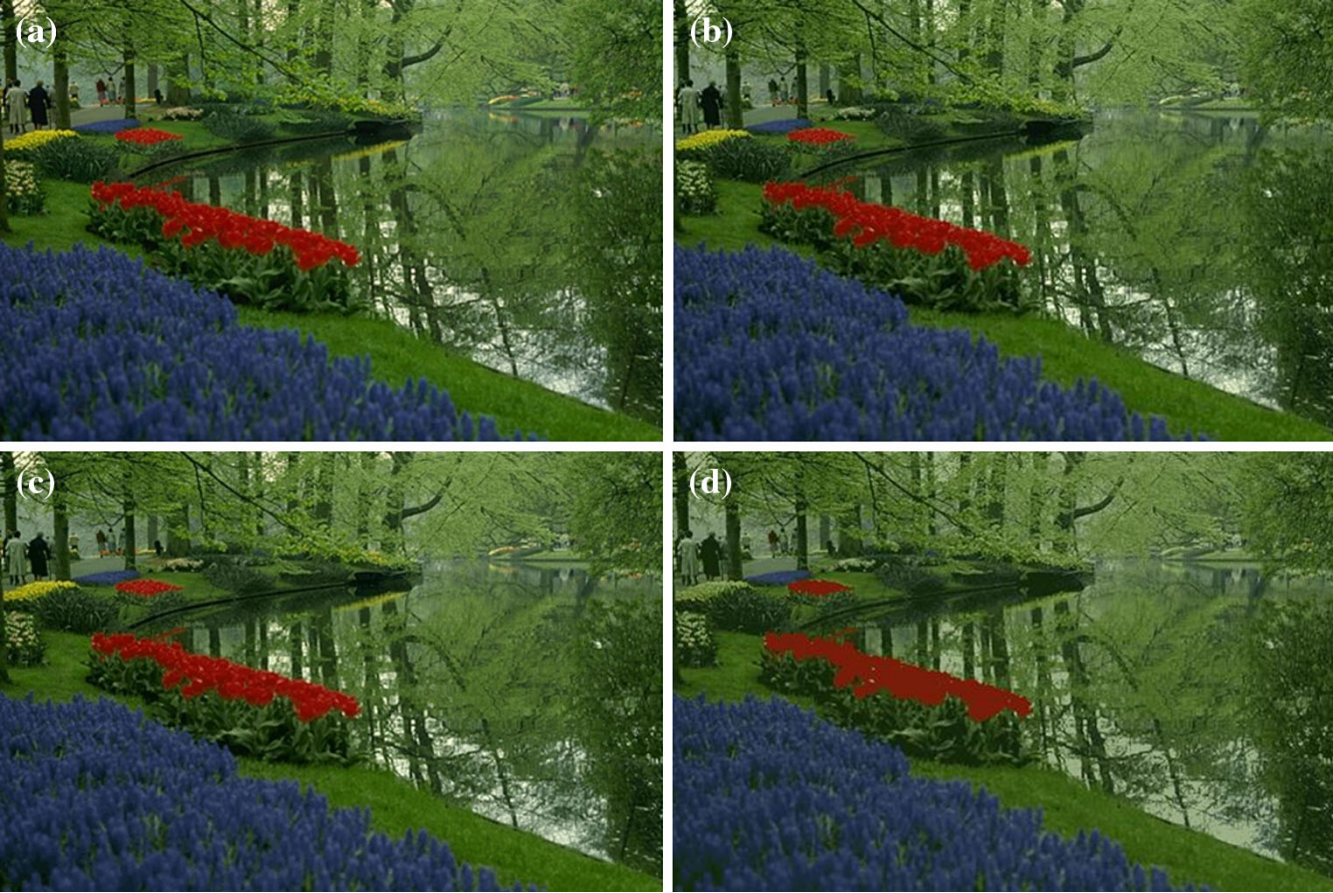
Reconstructed images with reference to the “river” image. **a** Original image. **b** Using colors achieved by the conventional SOM after 200 training epochs. **c** Using colors achieved by MFD-SOM after 20 training epochs. **d** Using colors achieved by the conventional SOM after 20 training epochs (color figure online)

By using color features extracted by both algorithms, reconstructed images with reference to the “river” image are presented in Fig. 8. Figure 8b presents the reconstructed image by the conventional SOM algorithm after 200 training epochs. Figure 8c shows the result image reconstructed by the MFD-SOM algorithm, and it is noteworthy that only twenty training epochs are used. Twenty training epochs are also set for the conventional SOM algorithm, and the corresponding result is shown in Fig. 8d.

From Fig. 8d, we can see that conventional SOM achieved poor representations of yellow and red colors. Due to strictly complying with the winner-take-all competitive principle, the conventional SOM always gives priority to learning features of principal components. Thus, given shorter training time, the conventional SOM may achieve unsatisfactory representations of those features of non-principal components in the training data. In contrast, even given shorter training time, MFD-SOM can still extract features with satisfactory quality, due to its learning being driven by training error.

### Learning behaviors of both algorithms

**Fig. 9:**
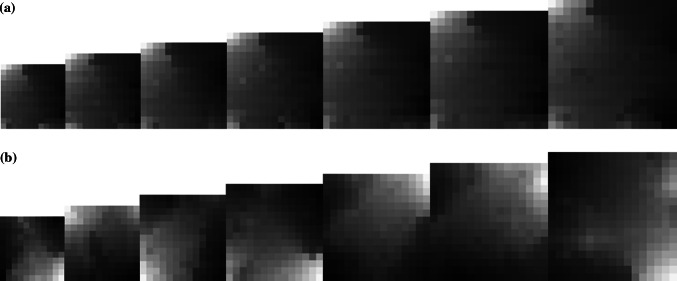
Sets of color feature maps of the “river” image achieved by both algorithms. **a** Maps achieved by the conventional SOM. **b** Maps achieved by the MFD-SOM (color figure online)

Map sizes ranging from 11 × 11 to 17 × 17 are set for both algorithms in order to extract color features from images in Fig. 5. As an example, color feature maps of the “river” image achieved by both algorithms are presented in Fig. 9, and they are ordered according to their map sizes.

From Fig. 9a, it can be seen that given more neurons, the conventional SOM gets more but redundant features. While Fig. 9b shows that numbers of all kinds of colors increase in a balanced way. Thus, theoretically, given more neurons, our algorithm can achieve features with higher quality because more candidate neurons can be utilized to store features of non-principal components in the training data.

Using above color features extracted by both algorithms, we got 14 reconstructed images with reference to the “river” image. The peak signal to noise ratio (PSNR) is applied to measure the quality of reconstructed images, and data are shown in Tables 1 and 2.

Comparison of PSNR data presented in Tables 1 and 2, it appears that both algorithms have their own advantages. In fact, as to the quality of a reconstructed image, which algorithm can get better score depends on the ratios of the non-principal components in the training data. If the ratios of the non-principal components in the training data are small, the conventional SOM will perform better in the evaluation. On the contrary, due to distortions of the reconstructed image to the original image are mainly caused by non-principal components, then our algorithm will win.

However, the PSNR data of the “river” and “penguin” images show that the scores of MFD-SOM far exceed the conventional SOM’s, while similar cases do not happen to the conventional SOM. It is worth noting that when the conventional SOM algorithm encounter similar cases, it will get bad scores because of the proportion of the non-dominant colors in images are considerable. In contrast, we can say that our algorithm is more robust in color feature extraction.

### Color extraction from image dataset

**Fig. 10:**
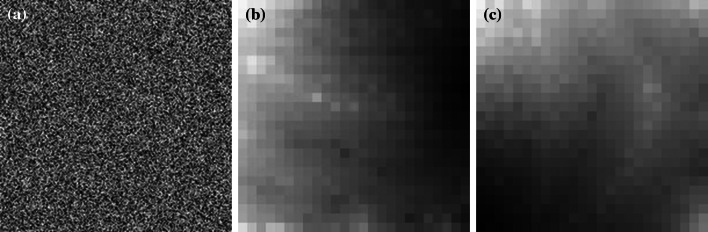
Demonstration of colors extracted from image dataset. **a** 40,000 color samples. **b** Color feature map purified by the conventional SOM for (**a**). **c** Color feature map purified by our algorithm for (**a**) (color figure online)

Total 436 images contained in the “Faces_easy” category of the Caltech-101 dataset [27] have been used in this experiment to extract colors from them. First, colors of each image were extracted by the MFD-SOM, where 11 × 11 neurons are used. Second, we sampled 40,000 colors with replacement from extracted colors, and they are shown in Fig. 10a. Finally, both algorithms using 25 × 25 neurons are employed to purify those 40,000 samples, and the results are presented in Fig. 10b, c.

Results shown in Fig. 10b, c are somewhat surprising because of a considerable number of colors are not directly relevant to human faces, such as blue and green colors. By reviewing of original images in the dataset, we can find out those colors have a large proportion in the image dataset.

### Computational performance of the proposed algorithm

We also implemented the conventional SOM algorithm in C language by using the same framework of MFD-SOM. Both algorithms with 11 × 11 neurons were used to extract colors from images in Fig. 5. Same values of initial training parameters used by MFD-SOM in Sect. 4.2 were used for both algorithms. Experiments were performed on a PC workstation (Intel Pentium Dual-Core CPU E5800 3.2 GHz and 2 GB RAM), and each image was processed 15 times by both algorithms, respectively. The performance data of both algorithms were calculated and presented in Table 3.

**Table 3:** Performance data of both algorithms

Image	Size of image	The conventional SOM			The MFD-SOM		
		Average number of updating weights	Average training time (s)	Average PSNR (dB)	Average number of updating weights	Average training time (s)	Average PSNR (dB)
River	481 × 321	3.642E+07	11.315	39.0595	7.440E+06	7.699	39.2094
Flower	283 × 213	1.737E+07	4.511	36.9017	3.340E+06	3.224	37.5074
Bird	267 × 400	2.518E+07	6.895	43.1459	4.951E+06	5.023	43.8589
Cloth	251 × 168	1.019E+07	2.707	43.1753	1.965E+06	1.766	46.4798
Girl	270 × 285	1.760E+07	4.909	40.6361	3.543E+06	3.388	41.4868
Penguin	238 × 300	1.737E+07	4.511	41.5407	3.340E+06	3.224	44.3566

Table 3 presents the number of updating weights, computational time, and PSNR data of images processed by both algorithms. As expected, MFD-SOM is always faster than the conventional SOM. It can be seen that the number of update operations of the MFD-SOM is about half of the conventional SOM used. Due to such operations as vector finding or matching are common to both algorithms, time saved by our algorithm reflects its learning efficiency. The PSNR data in Table 3 show that the quality of features extracted by the MDF-SOM is always better than the conventional SOM achieved, which demonstrates the necessity to improve the conventional SOM.

## Conclusions and discussions

In this paper, we present an improved SOM algorithm and its applications to color feature extraction. Compared with the conventional SOM algorithm, due to adoption of such strategies as dynamic neighborhood radius, dynamic learning rate, and a new way to update weights, the proposed algorithm can extract features with lower redundancy from dominant colors and achieve better representations of non-dominant colors in training images. Experimental results of color feature extraction from artificial datasets and benchmark image datasets demonstrate the characteristics of our algorithm. Besides those things, the proposed algorithm improves the performance on color feature extraction.

Quality of features extracted or data reduced by the conventional SOM algorithm heavily depends on the distribution of training data. However, for various reasons or purposes, sometimes, we are more concerned about how many features relevant to our application can be extracted from the training data and their quality. In this paper, a good attempt has been made by adopting new competitive rules in the proposed SOM algorithm to achieve robust color features from images. More rules involving filtering or selection of features can be introduced into the algorithm to extend its application.

## References

[1] Kohonen T (1998). The self-organizing map. Neurocomputing.

[2] Kohonen T (2001) Self-organizing maps, 3rd edn. Springer Ser Inf Sci 30:501

[3] Yin H (2008) The self-organizing maps: background, theories, extensions and applications. In: Computational intelligence: a compendium, vol 115. Springer, Heidelberg, pp 715–762

[4] Moschou V, Ververidis D, Kotropoulos C (2007). Assessment of self-organizing map variants for clustering with application to redistribution of emotional speech patterns. Neurocomputing.

[5] Ong SH, Yeo NC, Lee KH, Venkatesh YV, Cao DM (2002). Segmentation of color images using a two-stage self-organizing network. Image Vis Comput.

[6] Rasti J, Monadjemi A, Vafaei A (2011) Color reduction using a multi-stage Kohonen self-organizing map with redundant features. Expert Syst Appl 38(10):13188–13197

[7] Araújo ARF, Costa DC (2009). Local adaptive receptive field self-organizing map for image color segmentation. Image Vis Comput.

[8] Gdalyahu Y, Weinshall D, Werman M (2001) Self-organization in vision: stochastic clustering for image segmentation, perceptual grouping, and image database organization. Pattern Anal Mach Intell IEEE Trans 23(10):1053–1074

[9] Fyfe C, Barbakh W, Ooi W, Ko H (2008). Topological mappings of video and audio data. Int J Neural Syst.

[10] Vesanto J, Himberg J, Alhoniemi E, Parhankangas J (2000) SOM toolbox for Matlab 5. Tech Ber, Helsinki University of Technology

[11] Aoki T, Aoyagi T (2007). Self-organizing maps with asymmetric neighborhood function. Neural comput.

[12] Ontrup J, Ritter H (2001). Hyperbolic self-organizing maps for semantic navigation. Adv Neural Inf Process Syst.

[13] Sangole A, Knopf GK (2003). Visualization of randomly ordered numeric data sets using spherical self-organizing feature maps. Comput Graph.

[14] Nishio H, Altaf-Ul-Amin M, Kurokawa K, Kanaya S (2006). Spherical SOM and arrangement of neurons using helix on sphere. IPSJ Digit Cour.

[15] Schmidt CR, Rey SJ, Skupin A (2011). Effects of irregular topology in spherical self-organizing maps. Int Reg Sci Rev.

[16] Rauber A, Merkl D, Dittenbach M (2002). The growing hierarchical self-organizing map: exploratory analysis of high-dimensional data. Neural Netw IEEE Trans.

[17] Alahakoon D, Halgamuge SK, Srinivasan B (2000). Dynamic self-organizing maps with controlled growth for knowledge discovery. IEEE Trans Neural Netw.

[18] Fritzke B (1995). Growing grid—a self-organizing network with constant neighborhood range and adaptation strength. Neural Process Lett.

[19] Liu Y, Weisberg RH, Mooers CNK (2006). Performance evaluation of the self-organizing map for feature extraction. J Geophys Res.

[20] Kohonen T, Nieminen I, Honkela T (2009) On the quantization error in SOM vs. VQ: a critical and systematic study. Adv Self Organ Maps 5629:133–144

[21] DeSieno D (1988) Adding a conscience to competitive learning. Neural Netw IEEE Trans 1:117–124

[22] Papamarkos N, Atsalakis AE, Strouthopoulos CP (2002). Adaptive color reduction. IEEE Trans Syst Man Cybern B Cybern.

[23] Zagoris K, Papamarkos N, Koustoudis I (2007) Color reduction using the combination of the Kohonen self-organized feature map and the Gustafson-Kessel fuzzy algorithm. Mach Learn Data Min Pattern Recognit 4571:703–715

[24] Strong G, Gong M (2011). Similarity-based image organization and browsing using multi-resolution self-organizing map. Image Vis Comput.

[25] López-Rubio E, Ortiz-de-Lazcano-Lobato JM, López-Rodríguez D (2009). Probabilistic PCA self-organizing maps. Neural Netw IEEE Trans.

[26] Griffin G, Holub A, Perona P (2007) Caltech 256 object category dataset. Technical Report UCB/CSD-04-1366, California Institute of Technology

[27] Fei-fei L, Fergus R, Perona P (2007) Learning generative visual models from few training examples: an incremental bayesian approach tested on 101 object categories. Comput Vis Image Underst 106(1):59–70

